# Supplementary material for: Development of a computer-aided design software for the quantitative evaluation of aesthetic damage
Source: PLoS One. 2019 Dec 18;14(12):e0226322. doi: 10.1371/journal.pone.0226322 (PMC6919621; doi:10.1371/journal.pone.0226322)
Supplement: S1 Code site — 1. (ZIP) [file pone.0226322.s001.zip › [EESC_jr][Projeto]Relatorio_07_21/vendors/select2/docs/_includes/options/compatibility/query-function.html]

## Querying old data with `query`

Deprecated in Select2 4.0.
This has been replaced by another option and is only available in the
full builds of
Select2.

In the past, Select2 supported an option called `query` that
allowed for a custom data source to be used. This option has been replaced
by the `query` method on the
data adapter and takes a very similar set of
parameters.

Key
:   `query`

Value
:   A function taking `params` (including a `callback`)

Adapter
:   `DataAdapter`

Decorator
:   `Query`
